# Evaluation of Salivary and Serum micro RNA 146a, 200c and its Target Gene PTEN in Chronic Periodontitis Patients and their Response to Non-Surgical Periodontal Therapy

**DOI:** 10.2174/0122115366319964241020165218

**Published:** 2025-01-08

**Authors:** Jammula Surya Prasanna, Kunnel Apoorva

**Affiliations:** 1 Department of Periodontics. Panineeya Institute of Dental Sciences and Research Center. Road no. 5, Kamala Nagar, Dilsukh Nagar, Hyderabad, 500060, India

**Keywords:** Periodontitis, microRNA, PTEN, NSPT, Gene, RNA 146a, RNA 200c, saliva- serum

## Abstract

**Background:**

Periodontitis destroys the tooth's supporting structures and attachment apparatus. Local or systemic factors can cause it. Traditionally, diagnosis is based on clinical parameters that may not consistently reflect an accurate confirmation. Biochemical and genetic analyses can provide deeper insights. MicroRNAs (miRNAs) regulate the immune and inflammatory response to microbial pathogens. Detecting and evaluating miRNAs can be an important diagnostic parameter. This study aimed to assess the expression of miRNA 146a,200c, and its target gene PTEN to non-surgical periodontal therapy in serum and saliva.

**Materials and Methods:**

This interventional comparative study was conducted on 120 patients of both genders, ages between 35 and 55. Non-surgical periodontal therapy (NSPT) scaling and root planing were performed on all subjects, and their saliva and serum samples were collected before and after 8 weeks of NSPT. Quantitative rt-PCR (reverse transcriptase Polymerase Chain Reaction) analysis was conducted on all samples. The statistical analysis was done using SPSS version 22, and comparisons were made using paired t-tests, independent t-tests, and Pearson’s correlation coefficient. The statistical significance level was set at a ‘P’ value of less than 0.05.

**Results:**

It has been observed that there was a significant difference of miRNA in both serum and saliva samples 146a,200c, and the PTEN gene expression, from the beginning to 8 weeks. Significant variation was not observed when comparing the levels between serum and saliva.

**Conclusion:**

miRNA 146A, 200c, and PTEN genes are interrelated with periodontitis. We can consider them as future biomarkers of periodontal diseases.

## 1 INTRODUCTION

MicroRNAs (miRNAs) are a type of small, non-coding RNAs that are crucial in regulating gene expression. They can inhibit the expression of various genes, including those responsible for the host cell's immune responses against microbial pathogens. miRNAs regulate genes by targeting messenger RNA (mRNA) leading to mRNA degradation or translation inhibition [1-5]. Because of their stability and distinctive function, circulating miRNAs in body fluids such as serum and saliva have an advantage over other biomarkers such as proteins or bacterial products [6-9].

In the periodontium, miRNAs play a crucial character in the development and maintenance of periodontal homeostasis [10-14]. Specifically, miR-146a and miR-200c are members of the miRNA-146 and miRNA-200 families, respectively, and are intricate in regulating the immune response and inflammation [15-17] miR-146a acts as a negative regulator of inflammation by down-regulating adaptor proteins involved in inflammatory responses [18], whereas miR-200c regulates multiple signal pathways of pro-inflammatory factors and represses the expression and activity of Nuclear Factor kappa b (NF-kB) [17, 19].

Phosphatase and Tensin Homologue (PTEN) is a tumor suppressor gene that is expressed in periodontal tissues and plays a crucial role in tumorigenesis and bone remodeling [20]. The present study aimed to guesstimate the level of miR-146a, miR-200c, and PTEN in response to non-surgical periodontal therapy (NSPT). Understanding the underlying mechanisms of periodontal disease and developing new diagnostic and therapeutic strategies for managing periodontitis could be facilitated by studying the changes in miRNA expression and PTEN levels in response to NSPT.

## 2 MATERIALS AND METHODS

An interventional comparative study was steered with 120 patients of both genders aged 35-55. These patients were selected from the outpatient department of periodontics based on specific criteria explained in Fig. (**1**). Before the study, the protocol was explained to all participants, and written consent was obtained. The study received approval from the Institutional Ethical Committee and Review Board of *Panineeya Institute of Dental Sciences and Research Centre* (IEC No: PMVIDS&RC/IEC/PERIO/DN/O288-19) and was registered as trial number CTRI/2021/09/036784.

Patients with Stage II and Stage III periodontitis according to the AAP Classification 2017, [21] with 4-6mm pocket depth or 3-5 mm loss of attachment and those who have not received prior periodontal therapy within the preceding six months were included. The study excluded certain groups of patients, such as those who had taken antibiotics or anti-inflammatory drugs in the past 3 months, pregnant or breastfeeding women, individuals with medical conditions that could affect the study results, smokers, tobacco users, heavy drinkers, those undergoing high-dose steroid therapy, radiation or immunosuppressive therapy. Individuals with a history of genetic disorders, mental challenges, or uncooperative behaviour were excluded from the study, and those who did not follow oral hygiene instructions or attend follow-up appointments.

Clinical parameters assessed were Sulcus bleeding index (SBI) [22, 23], Plaque Index (PI) [24, 25], Probing Pocket Depth (PPD), and Clinical Attachment Level (CAL). Biochemical analysis of miRNA and gene was done using quantitative real-time PCR (q RT-PCR).

SBI index- Developed by Muehlemann HR and Son S in 1971. With a graduated periodontal probe four gingival units are screened systematically for each tooth. The labial and lingual marginal gingiva(M) and mesial and distal papilla (P). Add the total and divide by four will give the total score. 0- Healthy M no bleeding on probing, 1- Bleeding on probing, no colour change, no swelling of M, 2- Bleeding on probing, change in color, no swelling of M, 3- Bleeding on probing, change in color, slight swelling of M, 4- Bleeding on probing, change in color, obvious swelling of M, 5- Bleeding on probing, spontaneous bleeding, change in color, marked swelling with or without ulceration.

The Plaque Index (PI) was developed by Silness and Loe in 1964 to measure the amount of plaque on the gingival third of the teeth. It assesses the distofacial, facial, mesiofacial, and lingual surfaces of specific teeth, which are 16, 12, 24, 36, 32, and 44. The index uses a 0 to 3 scale: 0: No plaque;1: A film of plaque that can only be seen with a disclosing solution or probe; 2: Moderate accumulation of deposits visible to the naked eye;3: Abundance of soft material within the gingival pocket and/or on the tooth and gingival margin. To calculate the tooth score, sum the scores of the four areas and divide by 4. For individual total scores, divide by the number of teeth examined. For group total scores, divide by the number of individuals. The overall scores are categorized as: 0: Excellent;0.1-0.9: Good;1.0-1.9: Fair; 2.0-3.0: Poor 2ml blood was collected from the antecubital fossa by venepuncture, transferred to red-capped serum clot activator vacutainer tubes (KS Medical Pharmaceutical Company in Hangzhou City), and allowed to clot for 30min. Serum was obtained by centrifuging at 3000×g for 10min and then transferred to sterile Eppendorf tubes using droppers and labelled.

Patients were instructed to avoid using mouth rinses on the saliva collection day. Whole saliva samples were collected using passive drool into sterile disposable plastic tubes. These tubes were subjected to centrifugation to remove sediments. The supernatant saliva was subsequently transferred into Eppendorf tubes using sterile disposable droppers and labelled accordingly. All samples were then stored in Trizol solution at -20°C until the time of the assay. The samples were confidently collected at both baseline and 8 weeks post NSPT, performed by a highly skilled and experienced single expert.

Biochemical Analysis, miRNAs and genes were analyzed using q RT-PCR. RNA was First, the total RNA or messenger RNA (mRNA) was transcribed into complementary DNA (cDNA) using reverse transcriptase. Then, the cDNA is utilized as a template for the qPCR reaction. RNA was isolated from the samples using a Trizol reagent. Chloroform was added to distinguish the phases. The concentration of RNA was checked using electrophoresis and spectrophotometry. miRNA levels were measured by performing cDNA synthesis using a mRNA reverse transcription kit. Thermal cycling was then performed, and miRNA expression was analyzed relative to U6 miRNA (Tables **1a**-**b**).

PTEN expression levels were quantified using SYBR green chemistry, gene-specific primers, and appropriate cDNA dilutions. The 2-ΔΔCT method was used to calculate the expression levels, which were then normalized with control cells.

PCR cycling protocol, initial denaturation 95°C/2minutes, followed by 35 cycles of the following steps, denaturation 95°C/30 seconds, annealing 59.6°C/30 seconds (for miRNA 200C & U6 miRNA, for miRNA146C gene 57.9°C), extension 72°C/30 seconds followed by final extension Step-72 °C/10 minutes.

### 2.1 Agarose Gel Electrophoresis and Imaging

10 ml of PCR products of GAPDH, PTEN, U6, miRNA200C-5p, miRNA146a-5p, and 100 bp DNA ladder were loaded in wells of 2% agarose gel consisting of ethidium bromide. The gel was subjected to 80 V current for 30 to 45min. After the completion of the gel run, images were taken with the Chemi Doc imaging system (Bio-Rad).

### 2.2 Real-Time PCR

Once the amplification was confirmed by semi-quantitative PCR, quantitative real-time PCR was carried out. Diluted cDNA (1:6) was used to amplify target genes with gene-specific primers and were quantified using SYBR green chemistry. The expression levels of PTEN were normalized with GAPDH and that of miRNA200C-5p and miRNA146a-5p were normalized with U6 CT values.

**Fig. (2):**
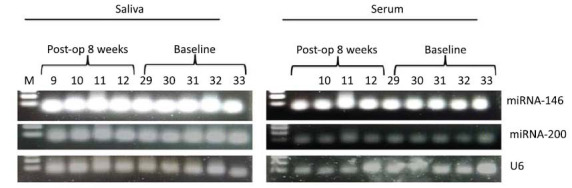
Micro RNA gel picture.

**Fig. (3):**
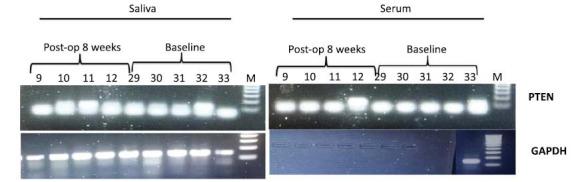
PTEN gene gel pictures.

**Fig. (4):**
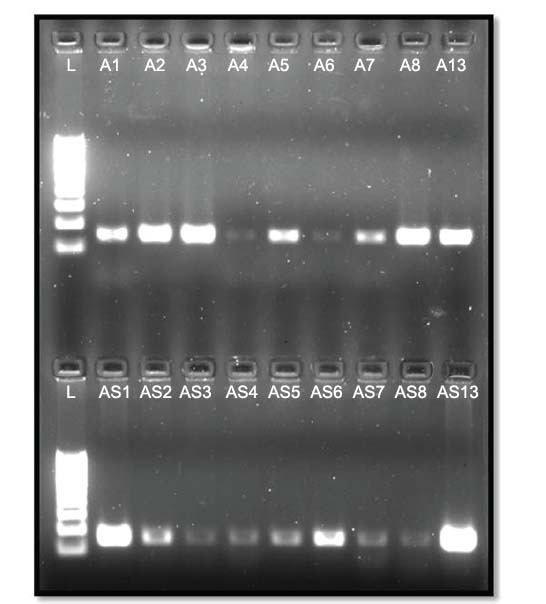
GAPDH PCR bands; Lane L is 100bp DNA ladder; Lanes: A1 to A8 & A13 and AS1 to AS8 & AS13 with 145bp GAPDH PCR bands.

**Fig. (5):**
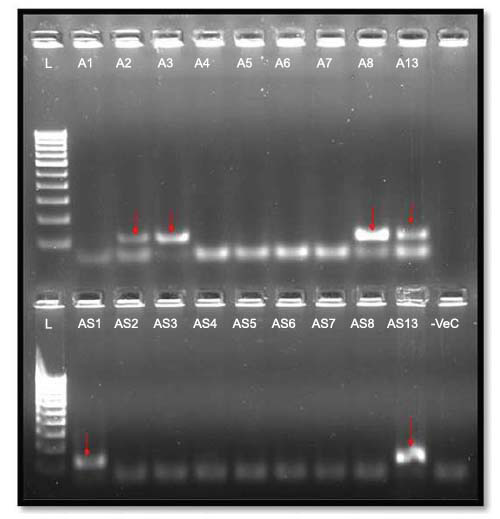
PTEN PCR bands; Lane L is 100bp DNA ladder; Lanes: A1 to A8 & A13 and AS1 to AS8 & AS13 with 106bp PTEN PCR bands (red arrow pointing to the target PCR bands).

**Fig. (6):**
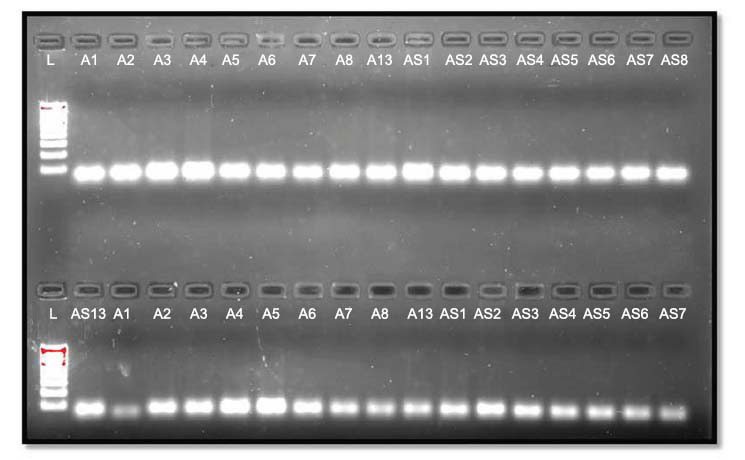
miRNA200C-5p PCR band; Lane-L is 100bp DNA ladder; Lanes: A1 to A8 & A13 and AS1 to AS8 & AS13 with miRNA200C-5p PCR bands and bottom lanes: A1 to A8 & A13 and AS1 to AS7 with miRNA146a-5p PCR bands.

**Fig. (7):**
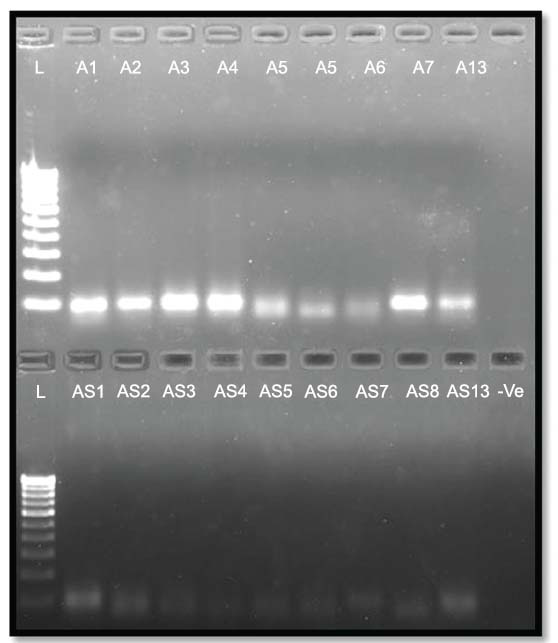
miRNA200C-5p PCR bands; Lane-L is 100bp DNA ladder;Lanes: A1 to A8 & A13 and AS1 to AS8 & AS13 with miRNA200C-5p PCR bands and bottom lanes: A1 to A8 & A13 and AS1 to AS7 with miRNA146a-5p PCR bands.

PCR products for all targets were run on a 2% agarose gel and imaged using the Bio-Rad Chemi Doc imaging system. Expression analysis was performed using the ImageJ software (Figs. **2-7**).

The Statistical Package for the Social Science (SPSS version 22, Armonk, NY: IBM Corp) was used to conduct the statistical analysis. The recorded values were evaluated statistically, and tests were performed at a 95% confidence interval level. The paired t-test was used for intra-group analysis, while the independent t-test was used for inter-group analysis. A statistically significant 'P' value was considered to be less than 0.05.

## 3 RESULTS

### 3.1 Comparison of Clinical Parameters at Baseline and 8 Weeks Post-Operatively

The Plaque Index (PI) levels were expressively higher (*p* <0.001) at baseline, measuring at 3.36 ± 0.90, compared to 0.61 ± 0.34 at 8 weeks post-operation. Similarly, the mean Sulcus Bleeding Index (SBI) levels were also suggestively higher at baseline (3.78 ± 0.88) than at 8 weeks post-operation (1.06 ± 0.60). The initial probing pocket depth (PPD) levels were also significantly higher at baseline (6.88 ± 0.87) compared to 8 weeks post-operation (2.27 ± 0.65) (*p*<0.001). Lastly, the baseline mean CAL was higher (4.23 ± 0.62) than the 8-week postoperative period (0.86 ± 0.71), which was also statistically significant (*p*<0.001) (Table **1**).

### 3.2 The Comparison of Serum and Salivary miRNA-146a at Baseline and 8 Weeks

The study found that the average levels of serum miRNA-146a were significantly higher at the beginning of the study (30.92 ± 1.39) than 8 weeks after the surgery (10.53 ± 1.13) (*p*<0.001). Similarly, salivary miRNA-146a was also higher at the start of the study (30.93 ± 1.41) than 8 weeks post-operative (10.37 ± 1.12). This difference was highly statistically significant (*p*<0.001). In addition, there was no statistically significant difference between the levels of miRNA-146a in serum and saliva, as evidenced by an independent t-test (*p*=0.964 and *p*=0.277 respectively) (Tables **2** and **3**).

### 3.3 Comparison of Serum and Salivary miRNA-200c from Baseline to 8 Weeks

The baseline average levels of serum miRNA-200c were significantly higher (31.04 ± 1.36) associated with the levels at 8 weeks post-operation (10.38 ± 1.09), with a highly significant difference (*p*<0.001). Similarly, the average of salivary miRNA-200c was also remarkably higher at baseline (30.94 ± 1.36) than at 8 weeks post-operation (10.40 ± 1.13), with a highly significant difference (*p*<0.001). No significant variation of miRNA-200c in serum and saliva at both baseline (*p*=0.569) and 8 weeks post-operation (p=0.907) (Tables **2** and **3**).

### 3.4 Comparison of Serum and Salivary PTEN at Baseline and 8 Weeks

The outcomes showed that the mean levels of serum PTEN were notably lower before surgery (10.48 ± 1.11) than 8 weeks after surgery (30.96 ± 1.44) (*p*<0.001). Similarly, the mean of salivary PTEN was also markedly lower before surgery (10.58 ± 1.10) than 8 weeks after surgery (30.97 ± 1.30) (*p*<0.001). However, there was no significant difference between the levels of PTEN in serum and saliva before surgery (*p*=0.52) or 8 weeks after surgery (*p*=0.934) (Table **3**).

## 4 DISCUSSION

Periodontal diseases, such as gingivitis and periodontitis, are common issues in oral health that can cause severe circumstances in the mouth. These diseases start with the formation of a harmful biofilm or plaque around the teeth, followed by an immune-inflammatory response from the host that worsens the disease [26]. An imbalance between the host and resident microbiota can occur due to weakened host responses or a surge in microbial challenge. Periodontal disease is characterized by alternating episodes of disease activity and quiescence, and if ignored, it can progress from mild inflammation to severe tissue destruction. The host's immune and inflammatory response to bacterial infection of the teeth causes the disease [27]. miRNAs, along with various other factors, play a critical role in driving this host response and the progression of the disease. MicroRNAs are a group of small non-coding RNAs, about 22 bp in length, that regulate gene expression through post-transcriptional modifications. They bind to the 3′-untranslated region of a target messenger RNA (mRNA), leading to gene expression suppression either by degrading a target mRNA or by preventing its translation [28].

MicroRNAs are an epigenetic mechanism that can influence various cellular processes including cell growth, apoptosis, and differentiation. They play a crucial role in inflammatory responses and the development of diseases [29]. A single miRNA can control the expression of multiple genes, while several miRNAs can regulate the expression of one gene. Abnormal levels of miRNA expression are linked with various chronic and acute diseases, which can affect gene expression and cellular functions during disease progression. During immune and inflammatory responses, miRNAs can target inflammatory regulators and influence the intensity of the inflammatory response [30].

 miRNAs are short, non-coding RNA molecules conserved across most organisms and make up approximately 1% of the human genome. In mammals, over 50% of protein-coding genes are controlled by miRNAs, also present in various body fluids. Some miRNAs may have specific functions associated with the surrounding tissues. Periodontal diseases are mainly caused by bacteria, particularly Porphyromonas gingivalis (P.g). P.g has been found to regulate the expression of miRNAs in gingival epithelial cells (GECs) and fibroblasts, which may play a role in the development of the disease [31].

The role and impact of miRNAs have been extensively studied in both healthy and diseased dental tissues including gingival fibroblasts, periodontal ligament cells, alveolar bone cells, dental follicle cells, dental pulp cells, dental papilla, and pre-and secretory ameloblasts. These small molecules have been found to play significant roles in various aspects of dental health such as periodontal disease, tooth movement and eruption, dental pulp physiology and pathology, differentiation of dental cells, and enamel mineralization [13]. Furthermore, numerous miRNAs have been recognized to be involved in the molecular pathways underlying periodontal inflammation [32].

Periodontal disease is a condition in which oral bacteria and inflammation disrupt the normal functioning of oral epithelial and immune cells and cause a dysregulation in the expression of miRNA in these cells. miRNA is produced by immune and non-immune cells within the cell and released into the extracellular environment, including extracellular fluids [33].

Two processes regulate bone - osteoblastic bone formation and osteoclastic bone resorption. These processes work together to maintain healthy bones. However, in periodontal disease, excessive bone resorption occurs, leading to the destruction of alveolar bone. miRNAs, small molecules, have a significant impact on osteoclast differentiation and function. Many different miRNAs have been recognized as having an impact on this process [10, 34]. It has been found that the function of miR-146a is up-regulated and this is NF-kβ dependent. The miR-146a targets TLR4 and IL-1, which helps to reduce cytokine production and regulate B-cell development. On the other hand, the miR-200c is down-regulated and has multiple functions including targeting TLR-4, inducing osteoclastogenesis, inhibiting endothelial cell differentiation, and angiogenesis [35].

MiRNA-146a is a member of the miR-146 family, which controls immune responses and inflammation. It's extremely expressed in blood cells and plasma has a negative correlation with pro-inflammatory cytokines, and is involved in inflammatory diseases. It targets the NF-kβ pathway, down-regulating key signaling adapter molecules [36] miR-146a suppresses toll-like receptor-2-mediated inflammation in keratinocytes and macrophages. It also regulates toll-like receptor sensitivity to prevent excessive inflammation. In addition, miR-146a is important in B-cell development and acts to prevent autoimmunity. It is expressed at low levels in naive T cells and is more abundant in memory T cells, with its expression induced upon TCR stimulation [37, 38].

The miR-200 family is known to regulate genes responsible for various dental epithelial cell lineages, playing crucial roles in both tooth development and pathological conditions within the oral cavity, including oral cancer and periodontitis. Studies have shown that miR-200 is abundantly expressed in human gingival keratinocytes and is implicated in downregulating NF-κB activation through TLR-4 signaling, potentially impacting the host's innate defenses against microbial pathogens. Additionally, miR-200c has been found to reduce LPS-induced pro-inflammatory mediators in human gingival fibroblasts and to effectively mitigate alveolar bone resorption in a rat experimental periodontitis model. Furthermore, it has demonstrated its ability to alleviate periodontal inflammation in obese mice with periodontitis [17, 39, 40].

Phosphatase and tensin homolog (PTEN) are critical regulators of tumorigenesis, bone remodeling, and cellular processes like apoptosis, autophagy, migration, proliferation, and survival. It also plays an imperative role in regulating the inflammatory response. Under normal conditions, the PI3K/Akt/PTEN signaling pathway plays a crucial role in inflammation. PTEN limits PI3K activity and downstream Akt signaling blocks TNF-stimulated NF-kβ-dependent genes and regulates osteoclast activity through inflammatory factors like IL-1 [41].

Reports have shown that periodontitis can influence the miRNAs in periodontal tissue, which may also alter the miRNA profile in the oral cavity and systemic circulation [41]. This study aimed to assess the miRNA-146a, miR-200, and PTEN genes in the saliva and serum of patients with chronic periodontitis and their response to NSPT. The study results showed that the miRNA-146a was higher at baseline than after NSPT. However, there was no significant variation between miRNA-146a assessed in serum and saliva. These findings align with the outcomes of previous studies by Al-Rawi *et al.* [42] and Ongoz Dede *et al.* [43] suggesting that miRNA-146a in saliva is a reliable, non-invasive diagnostic and prognostic biomarker for monitoring periodontal health status. Additionally, Palideh *et al.* [44] discussed the role of miRNAs in the immunopathogenesis of periodontal disease, highlighting the potential of miRNAs as therapeutic targets for treating periodontal disease. Furthermore, Radovic *et al.* [45] concluded that miR-146a may serve as a possible novel biomarker for periodontitis in non-diabetic and type 2 diabetic patients.

A few studies have investigated the role of miR-200 in periodontitis patients. *Kalea et al.* [46] found that miR-200 was significantly higher in the gingiva of obese periodontitis patients, which supports the results of this study. Yu *et al.* [47] along with miR-200 family miR-200c, also demonstrated significant upregulation in the gingival crevicular fluid (GCF) of chronic periodontitis (CP) patients compared to healthy controls. These miRNAs demonstrated strong diagnostic value and displayed positive correlations with clinical parameters of CP severity. Ogata *et al.* [48] concluded that miRNAs are associated with chronic periodontitis lesions in the Japanese population. In contrast, Stoecklin-Wasmer *et al.* [49] found that patients with periodontitis had reduced levels of miR-200a and miR-200c in their gingival tissues compared to healthy gingiva. However, the present study shows contrary results, as it indicates increased levels of miR-200c in chronic periodontitis patients compared NSPT. Akkouch *et al.* [50] stated that overexpression of miR-200c reduced the actions of proinflammatory and osteoclastogenic mediators.

According to our study, patients with periodontitis had lower levels of PTEN at the beginning compared to 8 weeks after NSPT. This finding is consistent with a study conducted by Fu *et al.* [20] demonstrated a significant reduction of PTEN in a mouse model of periodontitis induced by ligature.

To the best of our knowledge, no prior studies have investigated the association between the serum and salivary levels of miRNAs 146a, 200c, and the PTEN gene, thus rendering this study unique in this regard.

## CONCLUSION

Upon comparing the clinical parameters PI, SBI, PPD, and CAL before and after treatment, statistically significant changes were observed (*p* < 0.001), indicating the efficacy of NSPT in reducing gingival inflammation. Saliva and serum samples were collected for the analysis of micro RNA 146a, 200c, and PTEN gene. The results were confirmed using Quantitative rt-PCR (Reverse Transcriptase Polymerase Chain Reaction). Both serum and saliva exhibited noteworthy alterations (*p* < 0.001) in all three parameters from baseline to 8 weeks post-treatment. However, the rt-PCR values did not show significant disparities between saliva and serum, both at baseline and at 8 weeks. PCR products for all targets were subjected to analysis using a 2% agarose gel and imaged with the Bio-Rad Chemi Doc imaging system. The expression analysis was conducted using ImageJ software.

Based on these findings, it can be inferred that miRNAs can serve as effective biomarkers locally and systemically. Within the scope of our study, miRNA 146a and 200c were found to be upregulated in the diseased state, while the PTEN gene was downregulated. However, controversy exists regarding the precise role of these miRNAs in inflammation. Further, longitudinal studies and advanced molecular diagnostics are warranted to address these controversies concerning the role of miRNAs in the progression and resolution of inflammation.

## Figures and Tables

**Fig. (1):**
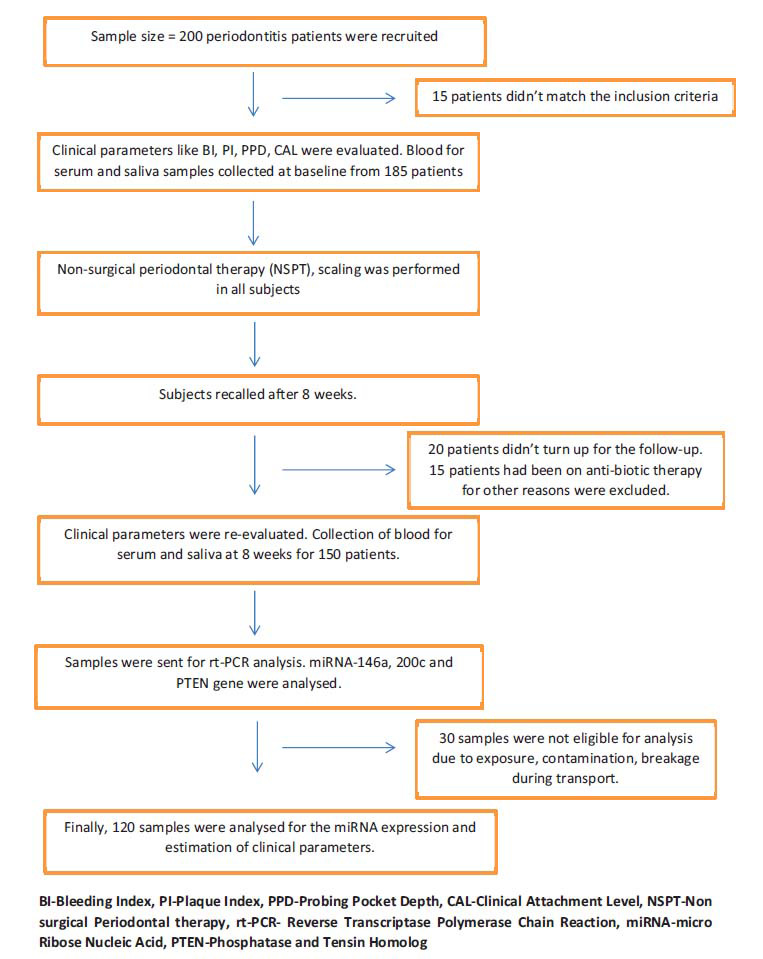
Patient selection criteria.

**Table 1a:** The primer sequences for the miRNAs were designed using the UCSC Genome Browser software.

**S. No**	**Target mRNA/miRNA**	**5’-3’ Sequence**
1	GAPDH Forward	GAGAAGGCTGGGGCTCATTTGC
2	GAPDH Reverse	TGGTGCAGGAGGCATTGCTGATG
3	PTEN Forward	GATGAGGCATTATCCTGTACACA
4	PTEN Reverse	CTCTTCAGATACTCTTGTGCTGT
5	U6 Forward	GCTTCGGCAGCACATATACTAAAAT
6	U6 Reverse	CGCTTCACGAATTTGCGTGTCAT
7	FP-miRNA200C-5p	CGTCTTACCCAGCAGTGTTTGG
8	FP-miRNA146a-5p	TGAGAACTGAATTCCATGGGTT
9	Universal reverse primer	AAAAAAGCGGCCGCTCTAGTT
10	Tagged oligo dT	AACTAGAGCGGCCGCTTTTTTTTTTTTTTTTTTTTTTTTT

**Table 1b:** Comparison of clinical variables at baseline and post-treatment using paired t-test.

**Variables**	**Baseline**		**Post**		**Difference**		**P Value**	**Significance**
	**Mean**	**SD**	**Mean**	**SD**	**Mean**	**SD**		
PI	3.36	0.90	0.61	0.34	2.75	0.96	<0.001	SIG
BI	3.78	0.88	1.06	0.60	2.72	1.07	<0.001	SIG
PPD	6.88	0.87	2.27	0.65	4.61	1.05	<0.001	SIG
CAL	4.23	0.62	0.86	0.71	3.37	0.91	<0.001	SIG

**Note:** PI-Plaque Index, BI-Bleeding Index, Pocket Probing Depth, CAL-Clinical Attachment Level. SD-Standard deviation, p- probability, sig- significance.

**Table 2:** Comparison between Serum and Saliva at baseline and post-treatment using paired t-test.

**Group**	**Variables**	**Baseline**		**Post (8Weeks)**		**Difference**		**P Value**	**Significance**
		**Mean**	**SD**	**Mean**	**SD**	**Mean**	**SD**		
Serum	146a miRNA	30.92	1.39	10.53	1.13	20.39	1.69	<0.001	SIG
	200c miRNA	31.04	1.36	10.38	1.09	20.66	1.45	<0.001	SIG
	PTEN Gene	10.48	1.11	30.96	1.44	20.48	1.53	<0.001	SIG
Saliva	146a miRNA	30.93	1.41	10.37	1.12	20.56	1.76	<0.001	SIG
	200c miRNA	30.94	1.36	10.40	1.13	20.54	1.65	<0.001	SIG
	PTEN Gene	10.58	1.10	30.97	1.30	20.38	1.63	<0.001	SIG

**Note:** miRNA-micro RNA, PTEN- Phosphatase and Tensin Homologue, SD-Standard deviation, P-Probability, SIG-Significance.

**Table 3:** Comparison of variables at baseline and post-treatment using independent t-test.

**Variables**	**Group**				**P Value**	**Significance**
	**Serum**		**Saliva**			
	**Mean**	**SD**	**Mean**	**SD**		
146a miRNA	30.92	1.39	30.93	1.41	0.964	NS
146a miRNA Post	10.53	1.13	10.37	1.12	0.277	NS
200c miRNA	31.04	1.36	30.94	1.36	0.569	NS
200c miRNA Post	10.38	1.09	10.40	1.13	0.907	NS
PTEN Gene	10.48	1.11	10.58	1.10	0.52	NS
PTEN Gene Post	30.96	1.44	30.97	1.30	0.934	NS

**Note:** miRNA-micro RNA, PTEN- Phosphatase and Tensin Homologue, SD-Standard deviation, P-Probability, NS-No Significance.

## Data Availability

The data and supportive information is available within the article.

## LIST OF ABBREVIATIONS

miRNAs: MicroRNAs
NSPT: Non-Surgical Periodontal Therapy
SPSS: Statistical Package for Social Sciences
P: Probability
mRNA: Messenger RNA
PTEN: Phosphatase and Tensin Homologue
AAP: American Academy of Periodontology
SBI: Sulcus Bleeding Index
PI: Plaque Index
PPD: Probing Pocket Depth
CAL: - Clinical Attachment Level
DNA: Deoxyribonucleic Acid
RNA: Ribonucleic Acid
GAPDH: Glyceraldehyde-3-phosphate Dehydrogenase
GECs: Gingival Epithelial Cells
NF-κB: Nuclear Factor Kappa B
GCF: Gingival Crevicular Fluid
CP: Chronic Periodontitis
